# Differential Effects of Omeprazole and Lansoprazole Enantiomers on Aryl Hydrocarbon Receptor in Human Hepatocytes and Cell Lines

**DOI:** 10.1371/journal.pone.0098711

**Published:** 2014-06-02

**Authors:** Aneta Novotna, Alzbeta Srovnalova, Michaela Svecarova, Martina Korhonova, Iveta Bartonkova, Zdenek Dvorak

**Affiliations:** Department of Cell Biology and Genetics, Faculty of Science, Palacky University, Olomouc, Czech Republic; Nihon University School of Medicine, Japan

## Abstract

Proton pump inhibitors omeprazole and lansoprazole contain chiral sulfur atom and they are administered as a racemate, i.e. equimolar mixture of S- and R-enantiomers. The enantiopure drugs esomeprazole and dexlansoprazole have been developed and introduced to clinical practice due to their improved clinical and therapeutic properties. Since omeprazole and lansoprazole are activators of aryl hydrocarbon receptor (AhR) and inducers of CYP1A genes, we examined their enantiospecific effects on AhR-CYP1A pathway in human cancer cells and primary human hepatocytes. We performed gene reporter assays for transcriptional activity of AhR, RT-PCR analyses for CYP1A1/2 mRNAs, western blots for CYP1A1/2 proteins and EROD assay for CYP1A1/2 catalytic activity. Lansoprazole and omeprazole enantiomers displayed differential effects on AhR-CYP1A1/2 pathway. In general, S-enantiomers were stronger activators of AhR and inducers of CYP1A genes as compared to R-enantiomers in lower concentrations, i.e. 1–10 µM for lansoprazole and 10–100 µM for omeprazole. In contrast, R-enantiomers were stronger AhR activators and CYP1A inducers than S-enantiomers in higher concentrations, i.e. 100 µM for lansoprazole and 250 µM for omeprazole. In conclusion, we provide the first evidence of enantiospecific effects of omeprazole and lansoprazole on AhR signaling pathway.

## Introduction

Proton pump inhibitors (PPIs) including omeprazole, lansoprazole, pantoprazole, rabeprazole and others are used in the treatment of gastroesophageal reflux disease (GERD), peptic ulcer disease as well as the eradication of *Helicobacter pylori* as a part of combination regimens. These drugs block the gastric H, K-ATPase by covalent binding at different cysteine residues and inhibit gastric acid secretion [1], [2]. In general, PPIs are weak bases administered most frequently orally in form of pro-drug. Their activation takes place in the acid space of the secretory canaliculus of the stimulated parietal cells resulting in the conversion to reactive sulfenamids [3], [4]. Omeprazole (OME) and lansoprazole (LAN) are substituted benzimidazoles that contain the asymmetric chiral sulfur atom in their chemical structure and therefore they exist in form R- and S-enantiomers. Initially, omeprazole was introduced to the market in 1989 as a racemic mixture. In 2001, an enantiopure drug Esomeprazole (S-enantiomer of OME) was developed, having improved metabolic properties, such as higher bioavailability in the majority of patients (extensive metabolizers and poor metabolizers) and lower interindividual variation as compared to racemic drug [5]–[7]. Similarly, lansoprazole was initially used as a racemate. Since R-enantiomer of lansoprazole, dexlansoprazole, constitutes more than 80% of circulating drug after oral administration of racemic drug, provides lower clearance and 5-fold greater systemic exposure than the S-enantiomer, FDA has approved dexlansoprazole in 2009 as an enatiopure drug for treatment of GERD [8], [9].

Omeprazole and lansoprazole are metabolized in the liver mainly by CYP2C19 with some contribution from CYP3A4 [10], [11]. However there are quantitative differences in stereoselective metabolism by human CYPs. Lansoprazole stereoselectivity seems to be mainly based on CYP3A4 selectivity in preference for the S-enantiomer, whereas for omeprazole stereoselectivity is based on both CYP3A4 preference for the S-enantiomer and CYP2C19 preference for the R-enantiomer [5], [8], [12]. In addition, omeprazole and lansoprazole have been shown to induce CYP1A genes in human hepatoma cells and primary human hepatocytes [13]–[15]. CYP1A genes are involved in the detoxification of xenobiotics such as drugs and environmental pollutants (polyaromatic hydrocarbons, dioxin-like compounds, polychlorinated biphenyls) as well as metabolic activation of these compounds. Induction of CYP1A1, CYP1A2 and CYP1B1 genes is mediated by aryl hydrocarbon receptor (AhR), which is a ligand-activated transcriptional factor that belongs to the bHLH/PAS (basic helix-loop-helix/PER ARNT Sim) family of transcriptional factors [16], [17]. Interestingly, molecular mechanism of CYP1A1/2 induction by benzimidazole proton pump inhibitors does not involve direct binding of the drugs to the AhR receptor, e.g. they are not ligands for AhR [18].

The aim of the current paper was to examine stereospecific effects of omeprazole and lansoprazole enatiomers on AhR-CYP1A signaling pathway. We measured transcriptional activity of AhR using gene reporter assay in transgenic cell line. The expression of CYP1A1/2 mRNA and protein was evaluated in human hepatoma cell line HepG2 and in primary human hepatocytes. Overall, current study provides the first evidence of enantiospecific effects of omeprazole and lansoprazole on the AhR signaling pathway.

## Materials and Methods

### Compounds and reagents

Dimethylsulfoxide (DMSO) and hygromycin B were purchased from Sigma-Aldrich (Prague, Czech Republic). 2,3,7,8-tetrachlorodibenzo-*p*-dioxin (TCDD) was from Ultra Scientific (RI, USA). S-omeprazole (S-OME), R-omeprazole (R-OME), rac-omeprazole (rac-OME), S-lansoprazole (S-LAN), R-lansoprazole (R-LAN) and rac-lansoprazole (rac-LAN) were purchased from Santa Cruz Biotechnology Inc. (Heidelberg, Germany). Luciferase lysis buffer was from Promega (Hercules, CA).

### Cell culture

Human Caucasian hepatocellular carcinoma cells HepG2 were purchased from European Collection of Cell Cultures (ECACC No. 85011430). Cells were cultured in Dulbecco's modified Eagle's medium (DMEM) supplemented with 10% of fetal bovine serum, 100 U/ml streptomycin, 100 µg/ml penicillin, 4 mM L-glutamine, 1% non-essential amino acids, and 1 mM sodium pyruvate. Cells were maintained at 37°C and 5% CO2 in a humidified incubator.

Primary human hepatocytes used in this study were obtained from two sources [19]: (i) from multiorgan donor HH52 (female; 60 years); the use of liver cells of donor HH52 was approved by “Ethical committee at the Faculty Hospital Olomouc”,and it was in accordance with Transplantation law #285/2002 Sb; “Ethical committee at the Faculty Hospital Olomouc” waived the authors from obtaining consent from the next of kin, regarding human hepatocytes obtained from liver donor HH52. (ii) long-term human hepatocytes in monolayer Batch HEP220770 (female; 35 years), Batch HEP220774 (female; 66 years) were purchased from Biopredic International (Biopredic International, Rennes, France). Cells were cultured in serum-free medium. Cultures were maintained at 37°C and 5% CO2 in a humidified incubator.

### mRNA determination and quantitative reverse transcriptase polymerase chain reaction (qRT-PCR)

Total RNA was isolated using TRI Reagent (Molecular Research Center, Cincinnati, OH, USA). cDNA was synthesized from 1000 ng of total RNA using M-MLV Reverse Transcriptase (Finnzymes, Espoo, Finland) at 42°C for 60 min in the presence of random hexamers (Takara, Shiga, Japan). qRT-PCR was carried out using LightCycler FastStart DNA MasterPLUS SYBR Green I (Roche Diagnostic Corporation, Prague, Czech Republic) on a Light Cycler 480 II apparatus (Roche Diagnostic Corporation). CYP1A1, CYP1A2 and GAPDH mRNAs were determined as described previously [20]. Measurements were performed in triplicates. Gene expression was normalized to GAPDH as a housekeeping gene.

### Protein detection and Western blotting

Total protein extracts were prepared from cells cultured on 6-well plates. Cells were washed twice with ice-cold PBS and scraped into 1 ml of PBS. The suspension was centrifuged (4500 RPM/5 min/4°C) and the pellet was resuspended in 150 µl of ice-cold lysis buffer (150 mM NaCl; 10 mM Tris pH 7.2; 0.1% (w/v) SDS; anti-protease cocktail, 1% (v/v) Triton X-100; anti-phosphatase cocktail, 1% (v/v) sodium deoxycholate; 5 mM EDTA). The mixture was vortexed and incubated for 10 min on ice and then centrifuged (15000 RPM/13 min/4°C). Supernatant was collected and the protein content was determined by the Bradford reagent. SDS–PAGE gels (10%) were run on a BioRad apparatus according to the general procedure followed by the protein transfer onto PVDF membrane. The membrane was saturated with 5% non-fat dried milk for 1 h at room temperature. Blots were probed with primary antibodies against CYP1A1 (goat polyclonal, sc-9828, G-18, dilution 1∶500), CYP1A2 (mouse monoclonal, sc-53614, dilution 1∶2000), actin (goat polyclonal; sc-1616, 1–19, dilution 1∶2000), all purchased from Santa Cruz Biotechnology (Santa Cruz, CA, USA). Chemiluminescent detection was performed using horseradish peroxidase-conjugated secondary antibodies (Santa Cruz Biotechnology) and Western blotting Luminol kit (Santa Cruz Biotechnology). The density of bands was measured by densitometry.

### Gene reporter assay and cytotoxicity assay

A stably transfected gene reporter cell line AZ-AHR, derived from human hepatoma HepG2 cells transfected with a construct containing several AhR binding sites upstream of a luciferase reporter gene, was used for assessment of AhR transcriptional activity [21]. Cells were incubated for 24 h with tested compounds and/or vehicle (DMSO; 0.1% v/v), in the presence or absence of TCDD (10 nM; AZ-AHR cells) or DEX (100 nM; AZ-GR cells). After the treatments, cells were lysed and luciferase activity was measured. In parallel, cell viability was determined by conventional MTT test.

### Statistics

Experiments in cell cultures were performed at least in four different cell passages. In each passage, treatments of cells were performed in triplicates. For measurement of luminescence (luciferase activity) and absorbance (MTT), triplicates from each sample were run. One-way analysis of variance followed by Dunnett's multiple comparison post hoc test or Student's *t* test was used for statistical analysis of data.

## Results

### Effects of omeprazole and lansoprazole enantiomers on transcriptional activity of aryl hydrocarbon receptor AhR in human gene reporter cell line AZ-AHR

**Figure 1:**
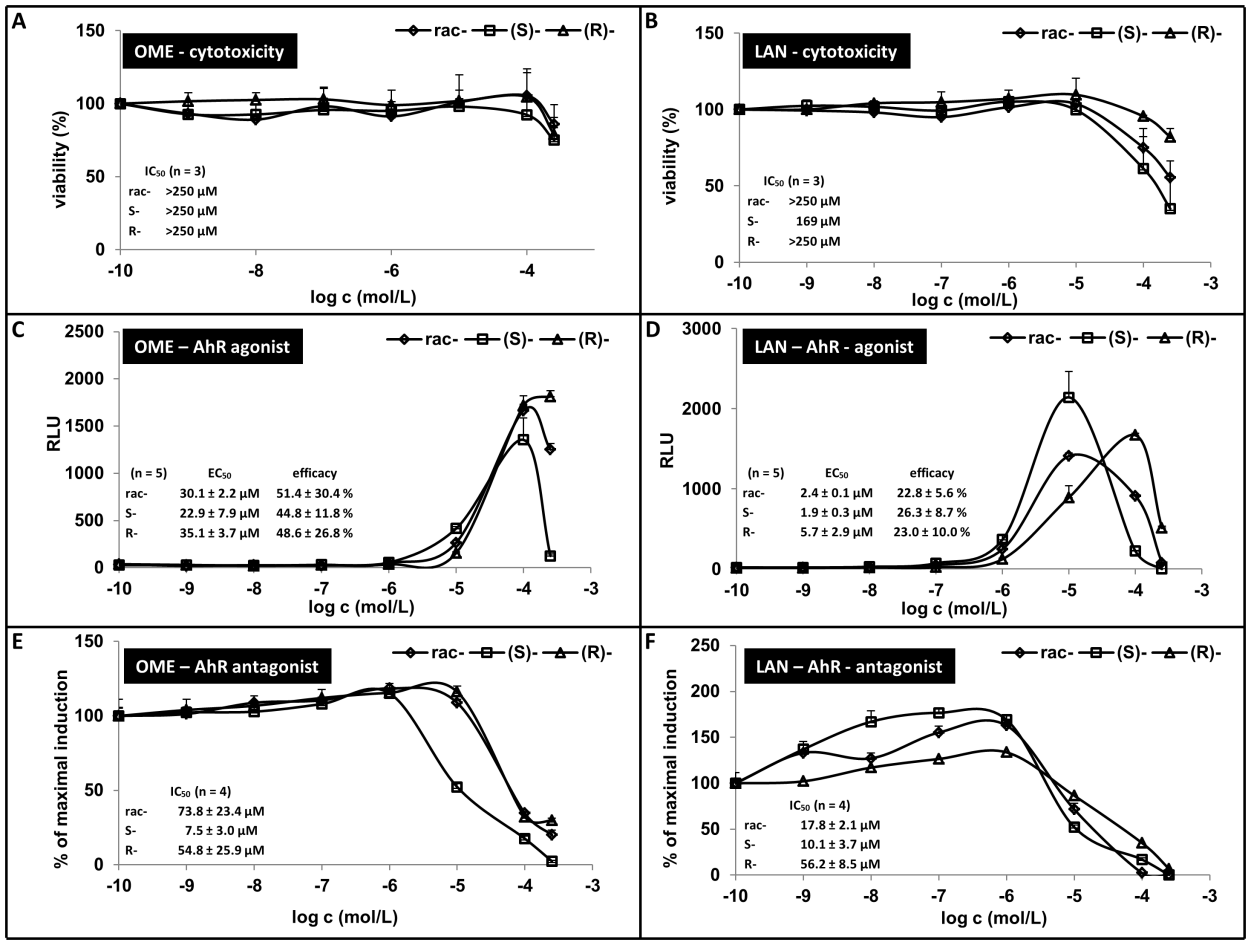
Effect of omeprazole and lansoprazole enantiomers on transcriptional activity of aryl hydrocarbon receptor AhR in human gene reporter cell line AZ-AHR. The cells were seeded in 96-well plates and stabilized for 16 h. **Panels A and B:** Cells were incubated for 24 h with S-OME, R-OME, rac-OME, S-LAN, R-LAN and rac-LAN at concentrations ranging from 10^−10^ M to 10^−4^ M. The vehicle was DMSO (0.1% v/v). After the treatment, MTT test was performed and absorbance was measured at 540 nm. Treatments were performed in triplicates. The data are the mean from experiments from six different passages of cells and are expressed as a percentage of viability of control cells. The values of IC_50_ were calculated and are indicated in a figure. **Panels C - F:** AZ-AHR cells were incubated for 24 h with S-OME, R-OME, rac-OME, S-LAN, R-LAN and rac-LAN at concentrations ranging from 10^−10^ M to 10^−4^ M in the absence (Panels C and D - *agonist mode*) or in the presence (Panels E and F- *antagonist mode*) of TCDD (5 nM). The vehicle was DMSO (0.1% v/v). After the treatments, cells were lysed and luciferase activity was measured. Treatments were performed in triplicates in five (*agonist mode*) or four (*antagonist mode*) independent cell passages. Representative gene reporter assays are showed. Data are expressed as a fold induction of luciferase activity over control cells (Panels C and D - *agonist mode*) or as a percentage of maximal induction attained by TCDD (Panels E and F- *antagonist mode*). The values of EC_50_ and IC_50_ were calculated and the average values are indicated in figures.

In the first series of experiments, the cytotoxicity of tested compounds was assessed in gene reporter cell line AZ-AHR. For this purpose, the cells were incubated for 24 h with S-OME, R-OME, rac-OME, S-LAN, R-LAN and rac-LAN at concentration ranging from 100 pM to 250 µM. The vehicle was DMSO (0.1% v/v). After the treatment, a conventional MTT test was performed. S-OME, R-OME and rac-OME were not cytotoxic in AZ-AHR cells at concentrations up to 250 µM. We found significant difference between cytotoxicity of lansoprazole enantiomers, with increasing toxicity in order R-LAN < rac-LAN < S-LAN (Figure 1A, Figure 1B).

Gene reporter assays were performed in two different experimental layouts. In *agonist mode*, cells were treated with increasing concentrations of tested compounds, and the half-maximal effective concentrations (EC_50_) were calculated, where appropriate. In *antagonist mode*, cells were incubated with increasing concentrations of tested compounds in combination with model agonist (TCDD; 5 nM), and half-maximal inhibitory concentrations (IC_50_) were calculated. An induction of AhR-dependent luciferase activity by 5 nM TCDD in five consecutive passages of AZ-AHR cells varied from 95-fold to 661-fold (average induction 354-fold), as compared to vehicle-treated cells. S-OME and rac-OME strongly, dose-dependently activated AhR up to concentration 100 µM with average EC_50_ of 22.9±7.9 µM and 30.1±2.2 µM, and average efficacy 44.8±11.8% and 51.4±30.4%, respectively. The luciferase activity declined at 250 µM concentration of S-OME and rac-OME, likely due to the AhR-unrelated effects. R-OME dose-dependently increased luciferase activity up to concentration 250 µM with an average EC_50_ of 35.1±3.7 µM and efficacy 48.6±26.8%. The efficacy was calculated at concentration of compounds of 100 µM and compared to 5 nM TCDD (Figure 1C). TCDD-inducible transcriptional activity of AhR was enantio-specifically and dose-dependently inhibited by all forms of omeprazole, with average IC_50_ values of 7.5±3.0 µM, 54.8±25.9 µM and 73.8±23.4 µM for S-OME, R-OME and rac-OME, respectively (Figure 1E). Lansoprazole dose-dependently increased luciferase activity up to concentration 10 µM (S-LAN, rac-LAN) and 100 µM (R-LAN). The average EC_50_ were 1.9±0.3 µM, 5.7±2.9 µM and 2.4±0.1 µM, for S-LAN, R-LAN and rac-LAN, respectively. The average efficacies were 26.3±8.7%, 23.0±10.0% and 22.8±5.6%, for S-LAN, R-LAN and rac-LAN, respectively (Figure 1D). At higher concentrations we found decrease of AhR-dependent luciferase activity likely due to the cytotoxicity or AhR-unrelated effects. Lansoprazole enantiospecifically decreased the TCDD-inducible luciferase activity in dose-dependent manner with IC_50_ values of 10.1±3.7 µM, 56.2±8.5 µM and 17.8±2.1 µM for S-LAN, R-LAN and rac-LAN, respectively (Figure 1F). Collectively, both OME and LAN showed enantiospecific effects on AhR transcriptional activity.

### Effects of omeprazole and lansoprazole enantiomers on CYP1A1 mRNA, protein and catalytic activity in human cancer cell line HepG2

**Figure 2:**
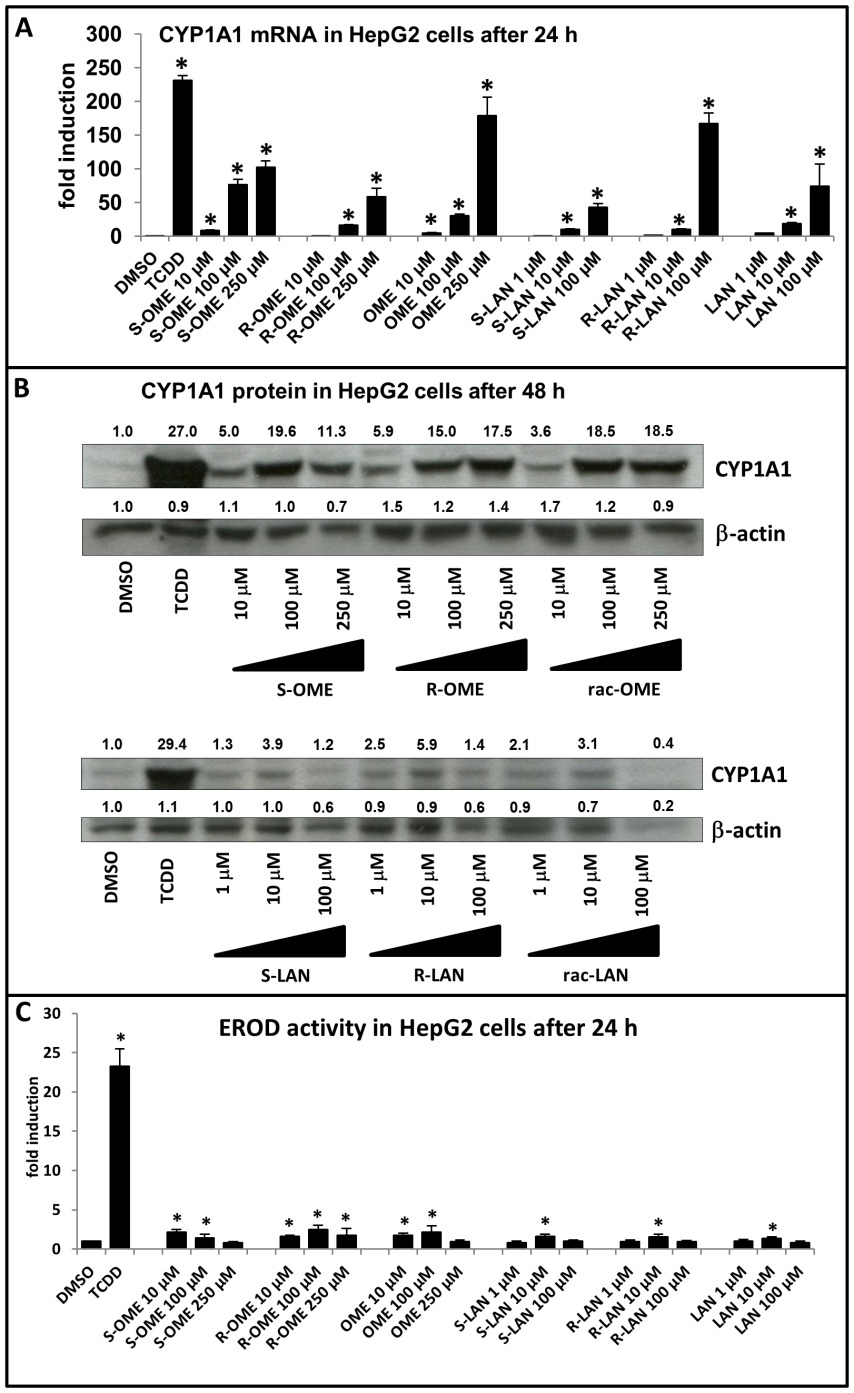
Effects of omeprazole and lansoprazole enantiomers on CYP1A1 mRNA, protein and catalytic activity in human cancer cell line HepG2. HepG2 cells were seeded in 6-well plates and stabilized for 16 h. All experiments were performed in three consecutive cell passages. Cells were incubated for 24 h (mRNA and EROD analysis) or 48 h (protein analysis) with TCDD (5 nM), vehicle (DMSO; 0.1% v/v), omeprazole (S-, R-, rac-; 10 µM, 100 µM, 250 µM) and lansoprazole (S-, R-, rac-; 1 µM, 10 µM, 100 µM). **Panel A:** Representative RT-PCR analyses of CYP1A1 mRNA are shown. The data are the mean ± SD from triplicate measurements and are expressed as a fold induction over vehicle-treated cells. The data were normalized to GAPDH mRNA levels. **Panel B:** Representative western blots of CYP1A1 protein are shown. Density of bands was quantified by densitometry and the values are indicated along with respective blots. **Panel C:** CYP1A1 catalytic activity (7-ethoxyresorufin-*O*-deethylase; EROD) was measured by spectrofluorometry with 530 nm excitation and 590 nm emission filters. Treatments were performed in triplicates. Average EROD data from three independent passages are showed. Data are expressed as a fold induction over vehicle-treated cells. An asterisk (*) indicates that the value is significantly different from the activity of vehicle-treated cells.

In next series of experiments, we tested the ability of omeprazole and lansoprazole enantiomers to induce the expression of prototypical AhR-responsive gene - CYP1A1. Human hepatoma HepG2 cells were treated with TCDD (5 nM), vehicle (DMSO; 0.1% V/V), S-OME, R-OME, rac-OME, S-LAN, R-LAN and rac-LAN at concentrations ranging from 1 µM to 250 µM for 24 h (mRNA expression, EROD activity) and 48 h (protein expression). Dioxin, a model activator of AhR and an inducer of CYP1A1 induced CYP1A1 mRNA approximately 230-fold as compared to vehicle-treated cells. All forms of omeprazole caused a concentration-dependent increase of CYP1A1 mRNA level. S-OME caused much stronger induction of CYP1A1 mRNA as compared to R-OME at all concentrations tested. However, the strongest induction of CYP1A1 mRNA was achieved by rac-OME at concentration 250 µM (approximately 180-fold induction) (Figure 2A). All forms of omeprazole induced CYP1A1 protein. Consistently with CYP1A1 mRNA data, S-OME caused higher induction of CYP1A1 protein than R-OME at concentration 100 µM. We observed decrease of CYP1A1 inducible protein level by S-OME but not by R-OME at concentration 250 µM likely due to cytotoxicity of S-OME after 48 h (observed in three consecutive passages) (Figure 2B). All forms of lansoprazole displayed a concentration-dependent increase of CYP1A1 mRNA level. R-LAN at concentration 100 µM caused the induction of CYP1A1 mRNA approximately four times stronger than S-LAN and two times stronger than rac-LAN (Figure 2A). All forms of lansoprazole caused only moderate induction of CYP1A1 protein; R-LAN showed stronger effect than S-LAN. Generally, we observed decrease of CYP1A1 inducible protein level at concentration 100 µM by all forms of lansoprazole, likely due to cytotoxicity of lansoprazole (Figure 2B). We also tested a capability of omeprazole and lansoprazole to induce catalytic activity of CYP1A1 in HepG2 cells (EROD assay). Cells were treated for 24 h with tested compounds, TCDD (5 nM) and vehicle (DMSO; 0.1% v/v). Dioxin induced EROD activity with the average increase of 22-23-fold. All forms of omeprazole induced catalytic activity EROD (6-9% of TCDD value), but with increasing concentration of omeprazole, activity diminished. Lansoprazole (R-, S-, rac-) significantly increased EROD activity only in concentration 10 µM (approx. 7% of TCDD value) (Figure 2C). Importantly, measurement of EROD activity comprises mixed effects of tested compounds in terms of enzyme induction, enzyme inhibition and possible cytotoxic effects. Overall, the effects of omeprazole and lansoprazole on CYP1A1 mRNA a protein expression in HepG2 were enantiospecific.

### Effects of omeprazole and lansoprazole enantiomers on CYP1A1 mRNA, protein and EROD activity in primary human hepatocytes

We examined a capability of omeprazole and lansoprazole to induce CYP1A1 and CYP1A2 mRNA and protein in primary human hepatocytes, which is a more physiological and metabolically competent cell model. Human hepatocytes were treated for 24 h or 48 h with TCDD (5 nM), vehicle (DMSO; 0.1% V/V) and tested compounds.

**Figure 3:**
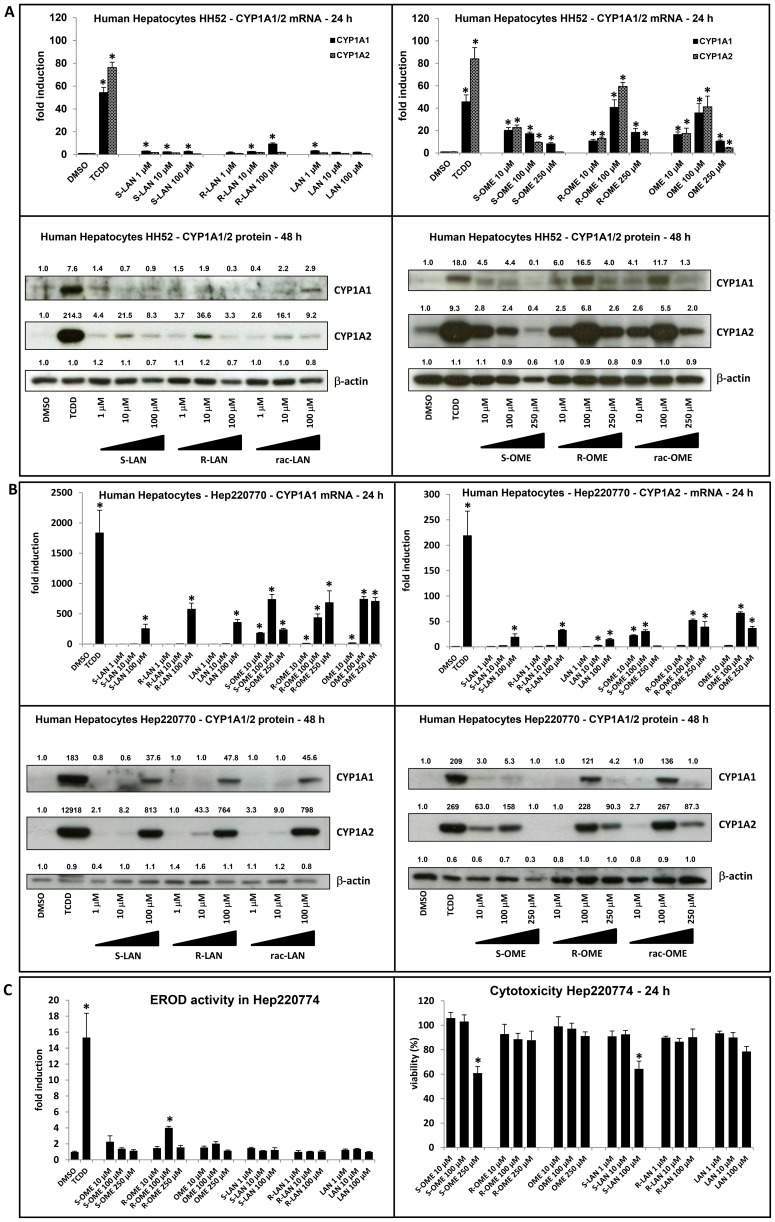
Effects of omeprazole and lansoprazole enantiomers on CYP1A1 mRNA, protein and EROD activity in primary human hepatocytes. **Panel A** and **Panel B:** RT-PCR analyses of CYP1A1 and CYP1A2 mRNA and western blots of CYP1A1 and CYP1A2 from two different cultures (HH52 and Hep220770) are shown. Human hepatocytes were incubated for 24 h (mRNA analysis) or 48 (protein analysis) with S-OME, R-OME, rac-OME, S-LAN, R-LAN, rac-LAN, TCDD and vehicle (DMSO; 0.1% v/v). RT-PCR data are the mean ± SD from triplicate measurements and are expressed as fold induction over vehicle-treated cells. Data were normalized to GAPDH mRNA levels. Density of bands in western blots was quantified by densitometry and the values are indicated along with respective blots. **Panel C:** EROD and cytotoxicity: Human hepatocytes (culture Hep220774) were treated for 24 h with S-OME, R-OME, rac-OME, S-LAN, R-LAN, rac-LAN, TCDD and vehicle (DMSO; 0.1% v/v). Upper bar graph: An activity of 7-ethoxyresorufin- O-deethylase (EROD) was measured by fluorescent spectrophotometry with 530 nm excitation and 590 nm emission filters. Treatments were performed in triplicates. The data are expressed as fold induction over the value from control cells. Lower bar graph: A conventional MTT test was performed and absorbance was measured at 540 nm. Treatments were performed in triplicates. The data are expressed as percentage of viability of control cells. An asterisk (*) indicates that the value is significantly different from the activity of DMSO.

Dioxin strongly induced CYP1A1 and CYP1A2 mRNAs and proteins in both human hepatocytes cultures. Induction profiles of CYP1A1/2 by omeprazole and lansoprazole enantiomers varied between individual human hepatocytes cultures. In culture HH52, CYP1A1 mRNA was weakly, but significantly induced only by all forms of lansoprazole, and the induction was strongest for R-LAN at concentration 100 µM (17% of TCDD induction). There was no induction of CYP1A2 mRNA by any form of lansoprazole (Figure 3A). In culture Hep220770, all forms of lansoprazole induced both CYP1A1 and CYP1A2 mRNA at concentration 100 µM. Consistently with data from culture HH52, R-LAN caused much higher induction of CYP1A1 mRNA (575-fold) than S-LAN (255-fold) and rac-LAN showed the combined effect of both enantiomers (358-fold) (Figure 3B). Similarly, the effect of R-LAN on CYP1A2 mRNA was approximately two times stronger as compared to S-LAN (Figure 3B). All forms of omeprazole caused induction of CYP1A1 and CYP1A2 mRNA in both cultures of primary human hepatocytes, but with different profile. In culture HH52, R-OME caused the highest induction of CYP1A1 and CYP1A2 at concentration 100 µM (89% and 71% of TCDD induction, respectively) (Figure 3A). In culture Hep220770, we observed strong induction of CYP1A1 mRNA by S-OME at concentration 100 µM (736-fold) (Figure 3B). R-OME dose-dependently increased CYP1A1 mRNA level with maximal induction at concentration 250 µM (684-fold). Rac-OME reached the maximal CYP1A1 induction at concentration 100 µM (740-fold) and slight decrease at concentration 250 µM (706-fold) (Figure 3B). Similar effect of omeprazole was found on induction of CYP1A2 mRNA (Figure 3B). In culture HH52, we found very faint or no induction of CYP1A1 protein after the treatment with any form of lansoprazole, while TCDD caused drastic increase of CYP1A1 protein. R-LAN and S-LAN at concentration 10 µM caused strong induction CYP1A2 protein (Figure 3A). In culture Hep220770, consistently with mRNA data, all forms of LAN induced CYP1A1 and CYP1A2 protein at concentration 100 µM (Figure 3B). In both cultures of primary hepatocytes, the effects of omeprazole enantiomers on CYP1A1 and CYP1A2 protein were in line with mRNA data. In culture HH52, R-OME at concentration 100 µM caused the highest induction of CYP1A1 and CYP1A2 protein as compared to S-OME and rac-OME (Figure 3A). In culture Hep220770, R-OME and rac-OME caused the strong induction of CYP1A1 protein at concentration 100 µM (Fig. 3B). All forms of omeprazole strongly induced CYP1A2 protein at concentration 100 µM and declined CYP1A2 protein level at concentration 250 µM (Figure 3B). Interestingly, S-OME but not R-OME and rac-OME at concentration 10 µM strongly induced CYP1A2 protein in both cultures of hepatocytes (Figures 3A and 3B).

We also tested capability of omeprazole and lansoprazole to induce catalytic activity of CYP1A1/1A2 enzymes and cytotoxicity in primary human hepatocytes. The cells were treated for 24 h with TCDD (5 nM), vehicle (DMSO; 0.1% V/V) and tested compounds. TCDD caused the induction of EROD activity approximately 15-fold while no significant induction of EROD activity was observed for any tested form of lansoprazole. Only R-OME induced EROD activity with maximum at concentration 100 µM and reached 27% of TCDD induction (Figure 3C). In cytotoxicity assay in human hepatocytes, we found that 250 µM S-OME and 100 µM S-LAN decreased viability of the cells down to 60% and 64%, respectively (Figure 3C).

## Discussion

The majority of biomacromolecules and molecules within living organism are chiral compounds, e.g. amino acids, carbohydrates, steroid hormones etc. Many exogenous compounds, including clinically used drugs, environmental pollutants or food constituents are also chiral compounds. Therefore the interactions between xenobiotics and structures in living systems are very often (nearly always) enantiospecific (stereospecific, stereoselective). The development of enantiopure drugs was logical output of these facts. Indeed, many drugs are racemic mixtures of enantiomers. Individual enantiomers may differ in their therapeutic activity both qualitatively and quantitatively. Therapeutically active enantiomer is called eutomer whereas its inactive counterpart is called dystomer. The ratio between activity of eutomer and dystomer is called eudysmic ratio. It is of value to use enantiopure drug, if eudysmic ratio largely differs from 1 (quantitative factor) or if one of the enantiomers exerts undesired or side effects (qualitative factor). Proton pump inhibitors (e.g. lansoprazole, om eprazole) are one of the most commonly prescribed drugs today. There are many studies on stereoselective metabolism and disposition of PPIs *in vitro* as well as *in vivo* [5], [22], [23]. Enantiopure formulations of omeprazole (S-; esomeprazole) and lansoprazole (R-; dexlansoprazole) were developed due to their improved pharmacokinetic properties as compared to the racemate. Since omeprazole and lansoprazole are activators of aryl hydrocarbon receptor (AhR) and inducers of CYP1A genes, we examined their enantiospecific effects on AhR-CYP1A pathway in human cancer cells and primary human hepatocytes. We performed gene reporter assays for transcriptional activity of AhR, RT-PCR analyses for CYP1A1/2 mRNAs, western blots for CYP1A1/2 proteins and EROD assay for CYP1A1/2 catalytic activity. We demonstrate that lansoprazole and omeprazole enantiomers display differential effects on AhR-CYP1A1/2 pathway. In general, S-enantiomers were stronger activators of AhR and inducers of CYP1A genes as compared to R-enantiomers in lower concentrations, i.e. 1–10 µM for lansoprazole and 10–100 µM for omeprazole. In contrast, R-enantiomers were stronger AhR activators and CYP1A inducers than S-enantiomers in higher concentrations, i.e. 100 µM for lansoprazole and 250 µM for omeprazole. At this moment, we can only speculate about the cause of enantiospecific effects of examined proton pump inhibitors on AhR-CYP1A1/2 signaling pathway *in vitro*. The possible mechanisms may include enantiospecific transmembrane transport (influx/efflux), stereoselective metabolism and stereospecific interaction with indirect AhR regulators such as tyrosin protein kinases.

In conclusion, in the current study we provide the first evidence of enantiospecific effects of omeprazole and lansoprazole on AhR signaling pathway. The results might have clinical significance.
